# Impact of the SARS-CoV-2 Pandemic in Candidaemia, Invasive Aspergillosis and Antifungal Consumption in a Tertiary Hospital

**DOI:** 10.3390/jof7060440

**Published:** 2021-05-31

**Authors:** Juan Vicente Mulet Bayona, Nuria Tormo Palop, Carme Salvador García, Begoña Fuster Escrivá, Mercedes Chanzá Aviñó, Pilar Ortega García, Concepción Gimeno Cardona

**Affiliations:** 1 Department of Microbiology and Parasitology, Consorcio Hospital General Universitario de Valencia, 46014 Valencia, Spain; nuriatormo@hotmail.com (N.T.P.); carme_salvador@hotmail.com (C.S.G.); begonafuster@gmail.com (B.F.E.); chanza_mer@gva.es (M.C.A.); concepcion.gimeno@uv.es (C.G.C.); 2 Department of Hospital Pharmacy, Consorcio Hospital General Universitario de Valencia, 46014 Valencia, Spain; ortega_mpi@gva.es; 3 Department of Microbiology and Ecology, University of Valencia, 46010 Valencia, Spain

**Keywords:** COVID-19, IFI, candidaemia, aspergillosis, antifungals, isavuconazole

## Abstract

In addition to the increase in fungal infections that has been observed in the last few decades, it has been reported that severe clinical COVID-19 can increase the risk of invasive fungal infections. The main objective of this study was to evaluate if there had been an increase in candidaemia and invasive pulmonary aspergillosis (IPA) cases since the onset of the SARS-CoV-2 pandemic. Data were retrospectively collected from April 2019 to March 2021, from patients admitted to Consorcio Hospital General Universitario de Valencia (Spain). A total of 152 candidaemia cases (56 of which were due to *Candida auris*) and 108 possible IPA cases were detected. A great increase in candidaemia cases was produced during the first and the third epidemic waves of the SARS-CoV-2 pandemic (June 2020, and January 2021, respectively), while an increase in IPA cases was produced during the third wave. The 28-day mortality rates in patients affected by candidaemia and IPA increased in 2020 and 2021. *C. auris* has displaced the other *Candida* species, becoming the most isolated *Candida* species in blood cultures since the onset of the SARS-CoV-2 pandemic. Antifungal consumption increased in 2020 when compared to 2019, especially echinocandins, voriconazole and isavuconazole.

## 1. Introduction

SARS-CoV-2, the virus responsible for coronavirus disease 2019 (COVID-19), rapidly spread in 2020 around the world, causing a global health emergency, being officially declared a pandemic by the World Health Organization (WHO) on 11 March 2020 [1]. Among the large number of secondary conditions that can be derived from COVID-19, there is an increasing concern about bacterial, fungal and viral co-infections and superinfections occurring in hospitalized patients with COVID-19 [2,3,4,5,6,7]. Regarding fungal infections, they present a particular clinical challenge due to their high mortality, the lack of fungal vaccines, limited diagnostics and increasing antifungal drug resistance [8]. In addition to the increase in fungal infections that has been observed in the last few decades [8], it has also been reported that severe clinical COVID-19 can increase the risk of invasive fungal infections (IFI), such as invasive candidiasis, invasive pulmonary aspergillosis (IPA) or *Pneumocystis jirovecii* pneumonia [3]. In this context, we previously reported a *Candida auris* outbreak occurring in our hospital, which seemed to have become endemic, and we noted that the onset of the SARS-CoV-2 pandemic had increased the *C. auris* colonization and candidaemia cases [9,10]. Some authors also reported new *C. auris* outbreaks in critically ill COVID-19 patients and alerted that the SARS-CoV-2 pandemic might facilitate the transmission of nosocomial pathogens such as *C. auris* [11,12,13].

Previous studies have shown that IFIs result in significant costs to healthcare systems [14]. Among several direct costs derived from IFIs, one of the most important is antifungal use. In fact, echinocandins, posaconazole and isavuconazole (ISV) are some of the most expensive antimicrobials currently available in the market. Voriconazole or ISV is usually recommended for the treatment of IPA [15], while echinocandins are the first-line treatment of candidaemia, especially due to fluconazole-resistant species such as *C. auris* [16]. Furthermore, early prevention methods for fungal diseases include prophylaxis and empiric treatment with antifungals.

ISV is a second-generation triazole approved for the treatment of invasive mucormycosis (IM) and IPA, based on two main clinical studies: the SECURE trial, where ISV showed non-inferiority to voriconazole for the primary treatment of IPA with fewer drug-related adverse events [17]; and the VITAL trial, where ISV showed comparable efficacy to amphotericin B against IM [18]. Furthermore, ISV has demonstrated activity against other fungal species, such as *Candida* spp., *Cryptococcus* spp. or endemic fungi [19,20,21]. ISV has also been used to treat the multidrug-resistant *C. auris*, as some reports demonstrated that ISV displayed activity and greater synergy when tested with anidulafungin than seen with anidulafungin plus voriconazole against the most resistant *C. auris* clinical isolates [22]. 

The objectives of this study are, therefore, (a) to evaluate if there has been an increase in candidaemia and invasive aspergillosis cases since the onset of the SARS-CoV-2 pandemic in our hospital; (b) to actualize the status of the *C. auris* outbreak occurring in our hospital; and (c) to examine if there has been an increase in the use of antifungals used to treat those fungal pathogens.

## 2. Materials and Methods

Data were retrospectively collected from April 2019 to March 2021, from patients admitted to Consorcio Hospital General Universitario de Valencia, a 503-bed tertiary hospital which provides medical assistance to a population of around 400,000 people in Valencia, Spain. 

A SARS-CoV-2 case was considered a patient with a SARS-CoV-2-positive test admitted to the hospital or in the emergency area. SARS-CoV-2 was detected by RT-PCR from nasopharyngeal samples using various methods (LightMix^®^, Roche Diagnostics, Basel, Switzerland; Panther Fusion^®^ SARS-CoV-2 Assay, Hologic, Marlborough, Massachusetts, United States; Aptima^®^ SARS-CoV-2 Assay, Hologic; CerTest SARS-CoV-2, CerTest Biotec, Zaragoza, Spain; Xpert^®^ Xpress SARS-CoV-2, Cepheid, Sunnyvale, United States) depending on the availability of the commercial reagents and the requirements and urgency of the results.

A possible IPA case was considered any growth of *Aspergillus* spp. in lower respiratory samples (bronchoaspirate and bronchoalveolar lavage fluid (BAL)) or in pulmonary biopsies. For sputum, only samples with a significant growth (reaching second strike in the plate) were considered. Samples were cultured in Sabouraud-chloramphenicol agar plates (Becton, Dickinson and Company, Franklin Lakes, NJ, USA), and the identification was carried out by macroscopic and microscopic examinations, and by matrix-assisted laser desorption/ionization (MALDI-TOF; Bruker), if possible.

Candidaemia cases were identified following our laboratory protocols. Briefly, blood samples were incubated in BD Bactec^®^ FX (Becton, Dickinson and Company) for 5 days, or 14 days in the cases where fungal blood infection was suspected. When the sample flagged positive, and the Gram stain revealed the presence of yeasts, subculture was performed in chocolate, Sabouraud-chloramphenicol and CHROMagar^®^ Candida (Beckton-Dickinson) agar plates. The plates were incubated at 37 °C and were read at 24 and 48 h of incubation. Grown colonies were further identified by matrix-assisted laser desorption/ionization (MALDI-TOF; Bruker, Billerica, MA, USA), and susceptibility testing was performed by Sensititre^®^ ITAMYUCC (Thermo Scientific, Waltham, MA, USA). For active surveillance studies of *Candida auris*, pharyngeal and axillary–rectal swabs were collected at admission to the intensive care unit (ICU) and once a week until hospital discharge. The swabs were cultured in CHROMagar^®^ Candida media, and the plates were incubated at 37 °C and read at 24 and 48 h. Non-specific white/beige/pink colonies were further identified by MALDI-TOF, and susceptibility testing was performed by Vitek 2^®^ AST YS-08 (bioMérieux, Marcy-l’Étoile, France). In some cases, a real-time PCR for direct detection of *C. auris* from the swabs was performed [23]. 

Antifungal consumption was calculated as defined daily doses (DDD) per 100 occupied bed days (OBD), according to the Anatomical Therapeutical Chemical Classification (ATC) methodology and the WHO DDD values [24].

## 3. Results

The first case of SARS-CoV-2 in Spain was confirmed on 31 January 2020, and the first case in our region (Valencia, Spain) was confirmed on 27 February 2020. Three epidemic waves have been described in the evolution of the pandemic in Spain: the first wave during spring 2020, the second one after the lockdown restrictions, during late summer and autumn, and the third one after Christmas and New Year’s Eve festivities, with a maximum peak in January 2021. The second wave, however, seemed to be milder than the other two, while the third wave more dramatically affected our region.

### 3.1. SARS-CoV-2 and Candidaemia

**Figure 1:**
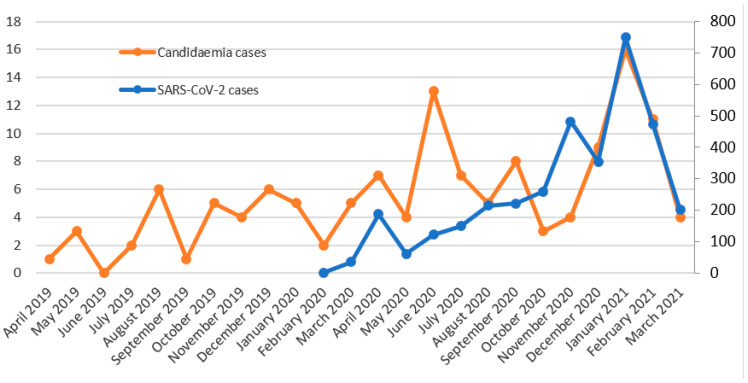
Temporal evolution of candidaemia cases (left axis) and SARS-CoV-2 cases (right axis).

A total of 152 candidaemia cases were detected, distributed as shown in Figure 1 and Table 1. Two clearly distinguishable peaks can be observed in the evolution of candidaemia cases (Figure 1). The first one was in June 2020, with 13 new candidaemia cases, two months after the peak of the first wave of COVID-19, and the second one was in January 2021, coinciding with the peak of the third wave. A total of 48.7% of the candidaemia cases were produced in patients admitted to the ICU. The 28-day mortality rates were 40.5%, 27.9% and 57.7% in 2019, 2020 and 2021 (until March), respectively.

**Figure 2:**
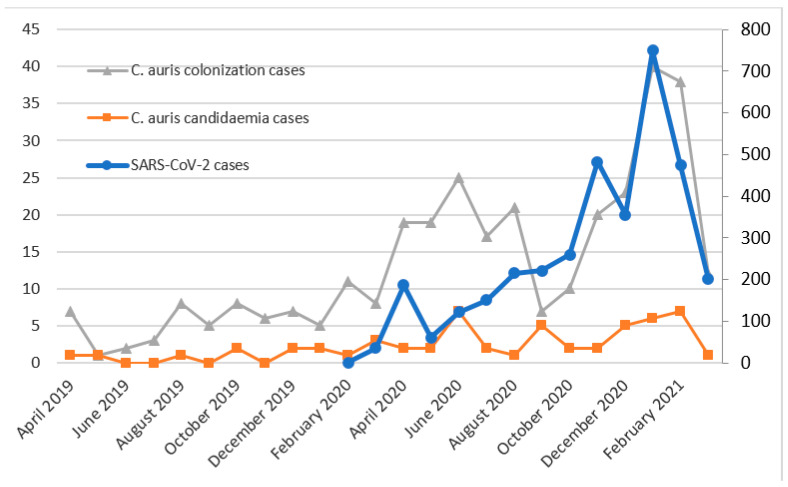
Temporal evolution of *C. auris* colonization and candidaemia cases (left axis) and SARS-CoV-2 cases (right axis).

Regarding *C. auris* candidaemia (Figure 2), 56 cases were detected. After the onset of the SARS-CoV-2 pandemic, two peaks can be observed, similar to those described for all Candida species candidaemia in Figure 1. This was expected, as *C. auris* has displaced the other Candida species, being the first cause of candidaemia in 2020 and in the first three months of 2021 (Table 1). After a first peak of *C. auris* candidaemia in July 2018, the outbreak seemed to be stable, with zero to two candidaemia cases every month, but with the SARS-CoV-2 pandemic, the cases increased again, reaching up to seven cases both in June 2020 and in February 2021. A total of 71.4% of the *C. auris* candidaemia cases were produced in patients admitted to the ICU. The 28-day mortality rates for *C. auris* candidaemia were 33.3%, 30.3% and 57.1% in 2019, 2020 and 2021 (until March), respectively. The curve of the new colonization cases is similar to that of the candidaemia cases, although in some cases, a peak in colonization becomes a peak in candidaemia the following month, as a portion of the colonized patients develop candidaemia between one and two weeks after the colonization is detected (e.g., the colonization cases increase in August 2020, and the candidaemia cases increase the following month). All *C. auris* isolates were resistant to fluconazole, and two isolates were also resistant to echinocandins. No other acquired resistance was observed in the other Candida species.

### 3.2. SARS-CoV-2 and IPA

**Figure 3:**
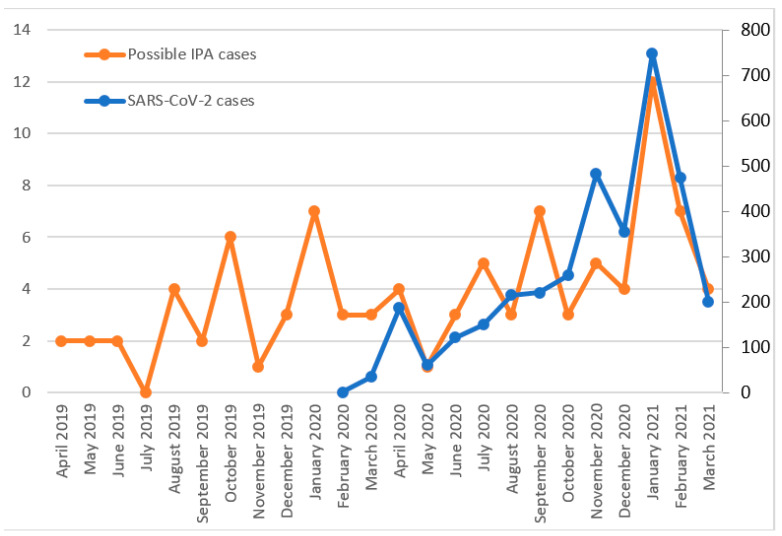
Possible IPA cases (left axis) and SARS-CoV-2 cases (right axis).

A total of 108 possible IPA cases were detected (37 in 2019, 48 in 2020 and 23 in January–March 2021). Although cases vary widely over the months, a large increase can be observed in January 2021, coinciding with the third wave of the SARS-CoV-2 pandemic (Figure 3). The isolated species were *Aspergillus fumigatus* (64.8%), *Aspergillus terreus* (13.9%), *Aspergillus niger* (13.9%), *Aspergillus flavus* (5.6%) and *Aspergillus nidulans* (1.8%). A total of 31.5% of the possible IPA cases were produced in patients admitted to the ICU. The 28-day mortality rates were 27.0%, 31.3% and 39.1% in 2019, 2020 and 2021 (until March), respectively.

### 3.3. SARS-CoV-2 and Antifungal Consumption

The antifungal consumption (DDD) increased in 2020, when compared to 2019, by 15% in the entire hospital (Table 2) and by 75% in the ICU (Table 3). The increase was remarkable, especially in the use of ISV, anidulafungin and micafungin in the entire hospital, and ISV, caspofungin, micafungin and amphotericin B in the ICU.

## 4. Discussion

The SARS-CoV-2 pandemic dramatically affected our region, especially the third epidemic wave. In this context, we noted an increase in the candidaemia and aspergillosis cases, which is in line with various works alerting that COVID-19, when it is severe, might increase the risk of invasive fungal infections [4,5]. For candidaemia, and also for *C. auris* candidaemia, two great increases were produced in June 2020, and January 2021, with the first peaks occurring two months after the first pandemic wave and the second peaks coinciding with the third wave. For IPA, a large increase was observed in January 2021. Although the crude mortality rates are difficult to evaluate since the SARS-CoV-2 virus was also involved, 28-day mortality rates were higher in January–March 2021, both for candidaemia (57.7%) and IPA (39.1%) cases, coinciding with the third epidemic wave of the SARS-CoV-2 pandemic. Additionally, the mortality rate for *C. auris* candidaemia (33.3%, 30.3% and 57.1% in 2019, 2020 and 2021, respectively) increased with respect to a previous work, where a mortality rate of 23.4% was reported [10].

Different ways in which COVID-19 can increase the risk of IFIs are discussed in the literature. First, severe COVID-19 patients require ICU admission, and they have all the classic risk factors for developing bacterial or fungal infections: mechanical ventilation, parenteral nutrition, broad-spectrum anti-bacterial treatment, central venous or bladder catheters, lymphopenia or corticosteroids [3]. Second, COVID-19 is associated with immune dysregulation itself, somehow facilitating invasion and infection by other pathogens [25]. Additionally, third, the SARS-CoV-2 pandemic has challenged the hospital settings globally, which might have led to an increase in the transmission of nosocomial pathogens such as carbapenem-resistant Enterobacteriaceae, methicillin-resistant *Staphylococcus aureus* (MRSA) or *C. auris* [13]. Chowdhary et al. discussed that, despite upgraded infection control measures to face SARS-CoV-2, the low compliance with the guideline of changing gloves and cleaning hands before and after each contact might result in cross-transmission between patients and in heavy contamination of the ICU environment, driven, in part, by the overoccupancy of beds and compromised practices of infection prevention control due to the pandemic [13]. Additionally, a relaxation of the measures to control *C. auris*, such as screening of patients for *C. auris* colonization and cohorting, occurred due to the higher workload of healthcare workers.

In September 2017, *C. auris* was isolated for the first time in our hospital, and we reported an outbreak which later became endemic [9,10]. After a peak in candidaemia cases occurred in July 2018, the occurrence of cases continued at a low rate, from zero to two every month, but we detected a new increase in June 2020, two months after the first wave of the SARS-CoV-2 pandemic. After the actualization of the data, it can be confirmed that the *C. auris* outbreak in our setting worsened due to the SARS-CoV-2 pandemic, detecting two peaks of candidaemia and colonization cases. Additionally, *C. auris* has displaced the other *Candida* species, becoming the most isolated *Candida* species in blood cultures since the onset of the SARS-CoV-2 pandemic. The overoccupancy of the ICU, where the outbreak was established, together with the higher workload of healthcare workers and the relaxation in compliance with the infection control measures might be the main causes. In October 2020, the increase in COVID-19 cases did not result in an increase in *C. auris* cases, which can be explained because the cohorting and screening for *C. auris* colonization were restored after the first wave. It is also remarkable that the increase in *C. auris* candidaemia during the third wave was not as high as expected based on the increase in COVID-19 cases, which can be explained by two reasons: first, the reinstatement of cohorting and screening for colonization; second, COVID-19 was under-diagnosed during the first wave, and therefore the peak of the first wave of COVID-19 would be higher.

Similar to the relation between the SARS-CoV-2 pandemic and the rise in candidaemia, there is a documented relation between COVID-19 patients and an increase in the cases of invasive pulmonary aspergillosis (IPA) [7]. IPA, the most severe manifestation of disease from *Aspergillus* spp., is associated with high mortality rates and is a prominent complication among those with profound immunosuppression, as well as those with structural lung damage, which is the case of COVID-19 critically ill patients [26]. In fact, it is well known that viral pneumonia increases patients’ susceptibility to bacterial and fungal superinfections, as influenza-associated pulmonary aspergillosis has complicated the clinical course of many critically ill patients with acute respiratory distress syndrome [27]. Furthermore, the administration of antibiotics results in a perturbation of the microbiome composition, which allows fungi to thrive and may predispose the host to invasive fungal infections once the immune system becomes impaired [8,28]. To discriminate between colonization and infection in patients with *Aspergillus*-positive respiratory samples is challenging, as diagnosis of IPA usually requires a comprehensive management strategy including a clinical assessment, high-resolution computerized tomography (CT) and bronchoscopy with BAL and fluid analysis and cytology to look for branching hyphae [29]. However, the same strategy may not be appropriate for immunocompetent critically ill patients, in whom the clinical signs and CT findings are less specific [30]. Still, although not all *Aspergillus*-positive cultures may represent IPA, a peak in isolations was produced in January 2021, coinciding with the third wave of the COVID-19 pandemic.

As a consequence of the increment in fungal disease, there has been an increased use of antifungals for treatment and prophylaxis, leading to an increased cost of healthcare [14]. In this context, rational use of antifungals and use of stewardship programs must be warranted, given the increasing threat of antifungal resistance, particularly in *Aspergillus* and *Candida* [31]. Azoles, especially ISV and voriconazole, and echinocandins showed a remarkable increase in 2020. The consumption of amphotericin B was also remarkable in the ICU, where a high number of candidaemia cases occurred, especially due to *C. auris*, and amphotericin B was used as the second-line treatment. Although the SARS-CoV-2 pandemic has increased fungal infections and antifungal consumption, it did not increase the antifungal resistance of isolates, except for the increase in the isolation of the fluconazole-resistant *C. auris* and two *C. auris* isolates which were also resistant to echinocandins.

Some works conclude that there is not enough evidence to support that co-infections and superinfections are more frequent in COVID-19 patients, but they also affirm that data are scarce and more prospective evidence is required [2,3,32]. In our study, an increase in the isolations of fungal pathogens can be clearly observed since the onset of the SARS-CoV-2 pandemic, although more investigations on the possible virus–fungus relationship are needed. The main limitation of this study is the fact that it is a descriptive and retrospective study. However, the study has the main strength of being performed in a tertiary hospital dramatically affected by the SARS-CoV-2 pandemic with an active *C. auris* outbreak. 

## 5. Conclusions

Since the onset of the SARS-CoV-2 pandemic, an increase in candidaemia and aspergillosis cases and, consequently, an increase in antifungal use have been observed in our setting, especially with ISV, voriconazole, echinocandins and amphotericin B. Additionally, a *C. auris* outbreak, which started in 2017 and seemed controlled, has worsened since June 2020, due to the pandemic. The cause might not be the virus itself alone but may also involve the overoccupancy of the ICU, the higher workload of healthcare workers and the relaxed compliance with the infection control measures. More prospective evidence is needed to determine if bacterial, fungal and viral co-infections and superinfections are more frequent in COVID-19 patients.

## Figures and Tables

**Table 1:** Etiology of candidaemia cases by year.

	2019 *n* (%)	2020 *n* (%)	2021 (Until March) *n* (%)
*Candida albicans*	18 (40.9)	15 (19.2)	11 (36.7)
*Candida auris*	9 (20.5)	33 (42.3)	14 (46.7)
*Candida glabrata*	8 (18.2)	9 (11.5)	1(3.3)
*Candida parapsilosis*	4 (9.1)	14 (17.9)	3 (1.0)
*Candida tropicalis*	3 (6.8)	4 (5.2)	1 (3.3)
Other species	2 (4.5)	3 (3.9)	0 (0.0)
Total	44	78	30

**Table 2:** Use of antifungals in 2019 and 2020 in the hospital.

Antifungal Group	DDD per 100 Bed Day 2019	DDD per 100 Bed Day 2020	Variation
Azoles	3.0642	3.8146	24.49%
Fluconazole	1.8801	2.0423	8.63%
Isavuconazole	0.0387	0.6185	1498.19%
Posaconazole	0.3225	0.1867	−42.11%
Voriconazole	0.8229	0.9671	17.52%
Echinocandins	1.3055	1.8783	43.88%
Anidulafungin	1.1228	1.6568	47.56%
Caspofungin	0.1221	0.1102	−9.75%
Micafungin	0.0606	0.1114	83.83%
Polyenes (Amphotericin B)	2.1227	1.7521	−17.46%
Total	6.4924	7.4450	14.67%

**Table 3:** Use of antifungals in 2019 and 2020 in the ICU.

Antifungal Group	DDD per 100 Bed Day 2019	DDD per 100 Bed Day 2020	Variation
Azoles	8.9511	15.7421	75.87%
Fluconazole	3.6841	2.4139	−34.48%
Isavuconazole	0.3914	7.6508	1854.74%
Posaconazole	0.3108	0.0539	−82.66%
Voriconazole	4.5648	5.6235	23.19%
Echinocandins	14.4162	18.4770	28.17%
Anidulafungin	13.1821	16.5693	25.70%
Caspofungin	0.5664	0.9188	62.22%
Micafungin	0.6677	0.9889	48.11%
Polyenes (Amphotericin B)	5.1808	15.7717	204.43%
Total	28.5481	49.9908	75.11%
